# The role of colchicine in the management of COVID-19: a Meta-analysis

**DOI:** 10.1186/s12890-024-03001-0

**Published:** 2024-04-20

**Authors:** Kholoud Elshiwy, Ghada Essam El-Din Amin, Mohamed Nazmy Farres, Rasha Samir, Mohamed Farouk Allam

**Affiliations:** 1 Department of Family Medicine, Faculty of Medicine, Ain Shams University, Cairo, Egypt; 2 Department of Community, Environmental and Occupational Medicine, Faculty of Medicine, Ain Shams University, Cairo, Egypt; 3 Department of Internal Medicine, Faculty of Medicine, Ain Shams University, Cairo, Egypt; 4 Department of Preventive Medicine and Public Health, Faculty of Medicine, University of Cordoba, 14004 Cordoba, Spain

**Keywords:** Coronavirus, COVID-19, SARS-CoV-2, Colchicine, Management, Meta-analysis, Ain Shams University

## Abstract

**Background:**

The Coronavirus disease 2019 (COVID-19) pandemic has robustly affected the global healthcare and economic systems and it was caused by coronavirus-2 (SARS-CoV-2). The clinical presentation of the disease ranges from a flu-like illness to severe pneumonia and death. Till September 2022, the cumulative number of cases exceeded 600 million worldwide and deaths were more than 6 million. Colchicine is an alkaloid drug that is used in many autoinflammatory conditions e.g., gout, familial Mediterranean fever, and Behçet’s syndrome. Colchicine inhibits the production of superoxide and the release of interleukins that stimulate the inflammatory cascade. Colchicine decreases the differentiation of myofibroblast and the release of fibrotic mediators including transforming growth factor (TGF-β1) that are related to the fibrosis. Moreover, colchicine has been used to traet viral myocarditis caused by CMV or EBV, interstitial pneumonia, and pericarditis resulting from influenza B infection. Additionally, colchicine is considered safe and affordable with wide availability.

**Objective:**

The aim of the current study was to assess the evidence of colchicine effectiveness in COVID-19 treatment.

**Methods:**

A comprehensive review of the literature was done till May 2022 and yielded 814 articles after ranking the articles according to authors and year of publication. Only 8 clinical trials and cohort studies fulfilling the inclusion criteria were included for further steps of data collection, analysis, and reporting.

**Results:**

This meta-analysis involved 16,488 patients; 8146 patients in the treatment group and 8342 patients in the control group. The results showed that colchicine resulted in a significant reduction in the mortality rate among patients received colchicine in comparison with placebo or standard care (RR 0.35, 95%CI: 0.15–0.79). Colchicine resulted in a significant decrease in the need for O2 therapy in patients with COVID-19 (RR 0.07, 95%CI 0.02–0.27, *P* = 0.000024). However, colchicine had no significant effect on the following outcomes among COVID-19 patients: the need for hospitalization, ICU admission, artificial ventilation, and hospital discharge rate. Among the PCR confirmed COVID-19 patients, colchicine decreased the hospitalization rate (RR 0.75, 95%CI 0.57–0.99, *P* = 0.042). However, colchicine had no effect on mortality and the need for mechanical ventilation among this subgroup.

**Conclusion:**

Colchicine caused a significant clinical improvement among COVID-19 patients as compared with the standard care or placebo, in terms of the need for O2, and mortality. This beneficial effect could play a role in the management of COVID-19 especially severe cases to decrease need for oxygen and to decrease mortality among these patients.

## Introduction

The Coronavirus disease 2019 (COVID-19) that was caused by coronavirus − 2 (SARS-CoV-2) has significantly impacted the healthcare and economic systems worldwide. The disease first began in Wuhan, China at the end of 2019. Then, it spread worldwide and became a pandemic. The clinical picture of the disease ranges from a flu-like illness to a massive inflammatory response and death [1]. In 2002 and 2003, there were outbreaks of severe respiratory distress syndrome in China. They occurred by SARS-CoV, another member of the coronavirus family. In 2012, another outbreak was documented in the Middle East and was caused by Middle East respiratory syndrome coronavirus (MERS-CoV) [2]. The current coronavirus is characterized by higher infectivity and geographical spread in comparison with both SARS and MERS. Therefore, COVID-19 was considered a significant global health threat that required robust efforts to minimize the burden of this pandemic [3].

The World Health Organization (WHO) announced that COVID-19 is a pandemic on 11 March 2020 [4]. Since then, the number of COVID-19 patients significantly increased. Till September 2022, the cumulative number of cases exceeded 600 million worldwide and deaths were more than 6 million [5].

The clinical manifestations of COVID-19 encompass symptoms such as fever, cough, dyspnea, malaise, or anosmia or ageusia, which can aid in early detection of the disease [6]. The primary mode of COVID-19 transmission is predominantly through exposure to infectious respiratory droplets from close contact with either symptomatic patients or asymptomatic carriers, as well as through aerosol particles that can remain suspended in the air for extended periods [7]. Additionally, indirect transmission through contaminated fomites, fecal excretion, environmental contamination, and fluid pollution has been documented, with viral viability reaching up to 72 hours after infecting surfaces [7, 8].

SARS-CoV-2 is a beta coronavirus that is a positive-stranded enveloped RNA virus. Similar to SARS-CoV and MERS-CoV, it is found in domestic and farm animals [9, 10]. The SARS-CoV-2 is characterized by spike proteins called S proteins. These proteins facilitate the viral infection through binding the S proteins and the angiotensin-converting enzyme 2 receptors (ACE2). These receptors are found in many tissues such as pneumocytes, enterocytes, renal cells, and endothelial cells [11]. SARS-CoV-2 causes marked dysfunction of the epithelial barrier and the endothelial cells of the pulmonary capillaries which triggers the migration and accumulation of inflammatory cells. This initiates the inflammatory cascade by both innate and cell-mediated immunity which significantly influences the alveolar-capillary oxygen transmission and the oxygen diffusion capacity [12].

In severe cases of COVID-19, fulminant inflammation, stimulation of the coagulation pathways, and consumption of the clotting factors occur in the form of a “cytokine storm”. This happens under the effect of many inflammatory mediators including interleukins, tumor necrosis factor-α (TNF-α), and interferon (IFN-γ). In addition, vasodilators such as bradykinin increase vascular permeability and result in pulmonary edema [13].

These mechanisms of cell damage represent a target for already existing medications that modulate the immune response. Based on its anti-inflammatory effects, colchicine has gained attention to be utilized in the management of COVID-19 patients. Colchicine is an alkaloid drug that is formed from a plant called “*Colchicum autumnale*”, also named “autumn crocus”. Colchicine is used in many autoinflammatory conditions e.g., gout, familial Mediterranean fever, and Behçet’s syndrome. Colchicine has an anti-inflammatory effect that is mediated through its binding to the tubulins and inhibiting the polymerization of microtubules. Microtubules are a key component of the cytoskeleton and are composed of tubulin heterodimers. These structures are important in different cellular functions including intracellular trafficking, cell shape, cell migration, and division [14]..

Colchicine inhibits the production of superoxide and the release of interleukin 1β and IL-6. Colchicine also prevents the inflammatory cascade by decreasing the production of inflammasomes that stimulate caspase-1 activation and release of interleukins such as interlukin1β and interleukin IL18 [15, 16]. Colchicine decreases the differentiation of myofibroblast and the release of fibrotic mediators including transforming growth factor (TGF-β1) [17, 18]. Moreover, colchicine has been used in cardiac conditions caused by a viral infection like myocarditis caused by CMV or EBV, interstitial pneumonia, and pericarditis resulting from influenza B infection. These different mechanisms greatly decrease the inflammatory response that represents a cornerstone in the pathophysiologic process of COVID-19. Besides the aforementioned effects of colchicine, its usage is considered safe and affordable with wide availability [19].

The ongoing impact of COVID-19 on all life aspects, the scarcity of effective treatments and the emergence of new virus variants resulted in the urgent need to repurpose the already existing drugs and to invent new therapeutic agents. This raised concerns about the effectiveness of colchicine in COVID-19 treatment and the possibility of providing an improvement in the clinical course of the disease.

The aim of the current study was to evaluate the efficacy of colchicine on different clinical outcomes including mortality, duration of COVID-19 illness till recovery, need for hospitalization, need for O2 therapy, need for ICU admission, and need for artificial ventilation.

## Methodology

### Criteria for considering studies for this meta-analysis

#### Types of studies

The review was restricted to Clinical Trials and Cohort Studies, which investigated the Colchicine administration in COVID-19 patients, versus standard treatment/placebo.

#### Types of participants

Participants were adult patients with the diagnosis of COVID-19. Patients were considered to have a definite diagnosis of COVID-19 if they were laboratory-confirmed using reverse transcription polymerase chain reaction (RT-PCR) and/or high-resolution CT chest with CO-RADS 4 or 5. All healthcare settings (community/primary care, hospital outpatient, or long-stay institutional) were considered eligible.

#### Types of interventions

Clinical trials and Cohort Studies were included. Colchicine was administered in COVID-19 patients, versus standard treatment/placebo.

#### Types of outcome measures

At least one of these outcomes was considered; Mortality, Duration of COVID-19 illness till recovery, Need for hospitalization, Need for O2 therapy, Need for ICU admission, and Need for artificial ventilation.

##### Inclusion criteria

(i) Cohort studies. (ii) Randomized and non-randomized clinical trials. Studies conducted on adult human subjects. (iii) Studies conducted on patients diagnosed with COVID-19 confirmed with positive reverse transcription polymerase chain reaction (RT-PCR) and/or high-resolution CT chest with CO-RADS 4 or 5. (iv) Studies conducted in all healthcare settings (community/ primary care, hospital outpatient or long-stay institutional). Studies published in Arabic, English, French or Spanish languages.

##### Exclusion criteria

Review, opinion studies, Case series, Studies conducted on animals.

##### Search strategy for identification of studies

Published studies and abstracts on the role of colchicine in the management of COVID-19 were identified through a comprehensive search of electronic databases that included PubMed (https://pubmed.ncbi.nlm.nih.gov/), ScienceDirect (www.sciencedirect.com), Scirus (www.scirus.com/srsapp), ISI Web of Knowledge (http://www.isiwebofknowledge.com), Google Scholar (http://scholar.google.com) and CENTRAL (Cochrane Central Register of Controlled Trials (http://www.mrw.interscience.wiley.com/cochrane/cochrane_clcentral_articles_fs.htm), using a combination of the following keywords: “Colchicine, COVID-19, Clinical Trail, Cohort Study”.

## Methods of the meta-analysis

### Locating and selecting studies

Abstracts of articles identified using the search strategy above mentioned were viewed, and articles that appeared to fulfil the inclusion criteria were retrieved in full. Data on at least one of the outcome measures was included in the study. Each article identified was reviewed and categorized into one of the following groups: Included: Randomized and non-randomized clinical trials, and Cohort studies that met the described inclusion criteria and those where it was impossible to tell from the abstract, title or MESH headings. Excluded: review, opinion studies, case series, and studies conducted on animals. When there was a doubt, a second reviewer (MFA) assessed the article, and a consensus was reached. The literature was reviewed till May 31, 2022 and yielded 814 articles after ranking the articles according to authors and year of publication. Only articles fulfilling the inclusion criteria were included (total 8 articles) for further steps of data collection, analysis, and reporting. The studies that met our inclusion criteria were Deftereos et al., Tardif et al., RECOVERY Collaborative Group, Lopes et al., Sandhu et al., Mareev et al., Brunetti et al. and Scarsi et al. [20–27]. All were in English and there were no available studies published in Arabic, French or Spanish language.

### Data extraction

A copy of each identified paper was obtained, and relevant data was abstracted by the first reviewer for a quantitative overview. We extracted the following study data from full-text articles: first author name, year of publication, study design, study location, eligibility criteria, sample size, age, sex, description of intervention and control groups, primary and secondary outcomes. In case of discrepancies or when the information presented in a study was unclear, abstraction by a second reviewer (MFA) was sought to resolve the discrepancy.

### Statistical considerations

Data were abstracted from every study in the form of a risk estimate and its 95% confidence interval. When a risk estimate and its 95% confidence interval were not available from the article, we calculated unadjusted values from the published data of the article, using the Epi Info 6 computer program version 6.04d.

Pooled estimates of relative risks were obtained by weighing each study by the inverse variance of the effect measure on a logarithmic scale. This approach to pool the results assumed that the study populations being compared were similar and hence corresponded to a fixed effect analysis. The validity of pooling the relative risks was tested (test of homogeneity) using chi square test.

A violation of this test suggested that the studies being pooled differed from one another. In the presence of significant heterogeneity of the effect measure among studies being compared, we performed a random effect analysis that was based on the method described by DerSimonian and Laird. The random effect analysis accounted for the interstudy variation. Because the test of homogeneity had low power, we reported the figures of the random effect analysis even with the absence of significant heterogeneity.

All statistical analyses for pooling the studies were performed on the MetaXL Software.

## Results

**Fig. 1:**
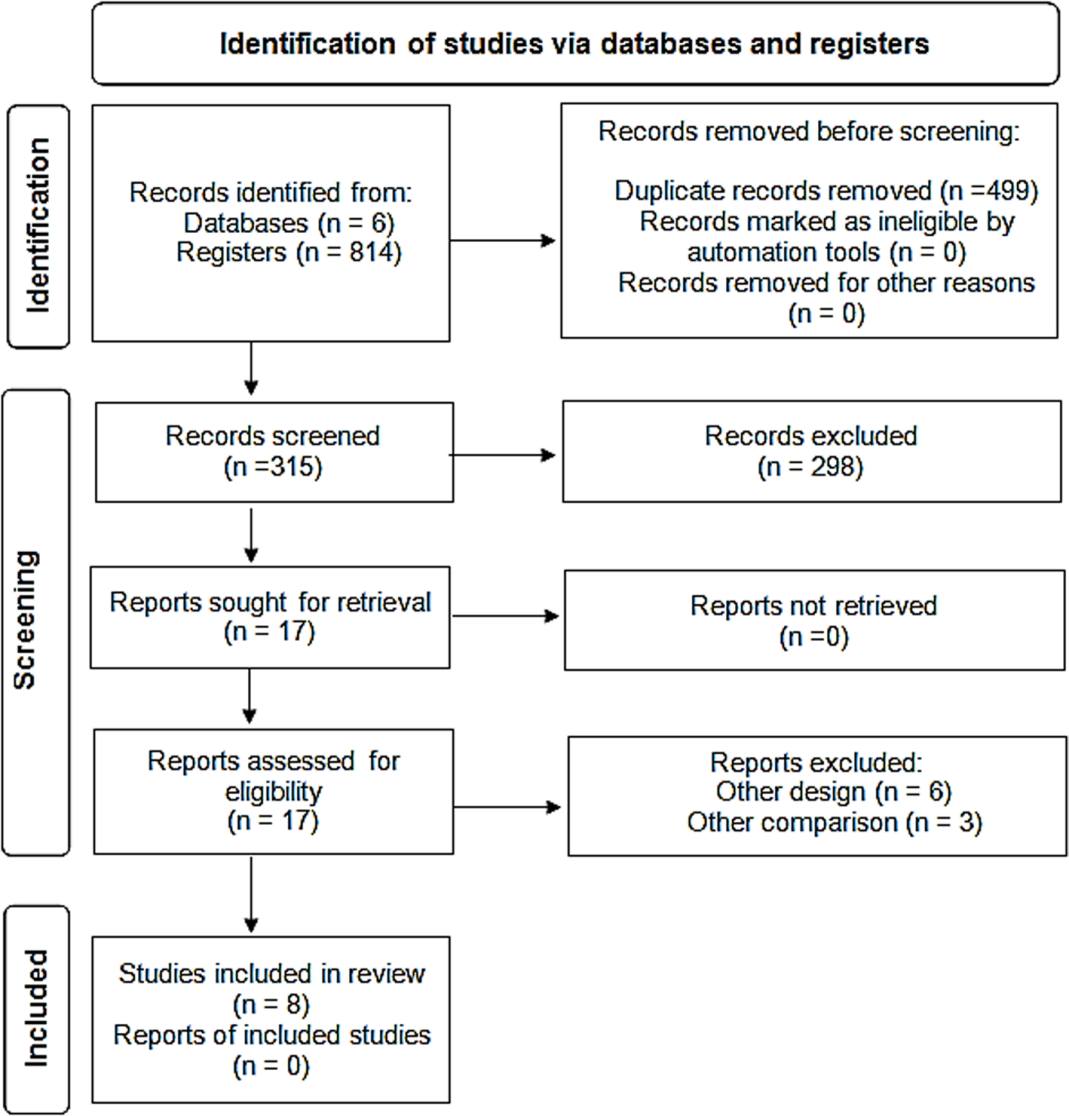
PRISMA flow diagram showing selection of studies. PRISMA; Preferred Reporting Items for Systematic Reviews and Meta-Analyses

In 6 databases, we identified 814 articles; 499 duplicates were removed. Out of the remaining 315 abstracts, we excluded 298 after screening. Thus, 17 full-text studies were assessed for eligibility and 9 were excluded. Finally, eight studies were included for further qualitative and quantitative analyses (Fig. 1).

### Characteristics of the included studies

Two studies were cohort (Brunetti et al. and Scarsi et al.) while the other studies were four randomized controlled clinical trials (Deftereos et al., RECOVERY Collaborative Group, Lopes et al., and Tardif et al.) and two non-randomized controlled clinical trials (Mareev et al., and Sandhu et al.).

Two studies were multicentre clinical trials (RECOVERY Collaborative Group, and Tardif et al.)***.*** The other six studies were conducted in Greece (Deftereos et al.), Brazil (Lopes et al.), the USA (Brunetti et al. and Sandhu et al.), Russia (Mareev et al.), and Italy (Scarsi et al.) [22–29].

The studies included both hospitalized and non-hospitalized COVID-19 patients, who were diagnosed either clinically or by laboratory diagnosis with PCR–RT testing and CT chest imaging (Table 1).

**Table 1:** Characteristics of included studies evaluating the efficacy of colchicine in COVID-19

Authors	Year of publication	Type of study	Study population	Participants (Colchicine/ Control)	Colchicine drug dose	Control	Outcomes
**Deftereos et al.**	2020	RCT	Hospitalized COVID-19 adult patients	55/50	Loading dose: 1.5 mg followed by 0.5 mg 60 minutes later. Maintenance dose: 0.5 mg twice daily until hospital discharge or for 21 days.	Standard treatment	Mortality, Mechanical Ventilation
**Tardif et al.**	2021	RCT	Non-hospitalized COVID-19 adult patients	2235/2253	0·5 mg twice per day for the first 3 days and then once per day for 27 days	Placebo	Mortality, Hospitalization, Mechanical Ventilation
**Lopes et al.**	2021	RCT	Hospitalized COVID-19 adult patients	36/36	0.5 mg 3 times daily for 5 days, then 0.5 mg twice daily for 5 days	Placebo	Mortality, ICU Admission, ICU length of stay
**RECOVERY Collaborative Group**	2021	RCT	Hospitalized COVID-19 adult patients	5610/5730	1 mg followed by 500 μg 12 hours later and then 500 μg twice a day for 10 days or until discharge	Standard care	Mortality
**Sandhu et al.**	2020	NRCT	Hospitalized COVID-19 adult patients	34/78	0.6 mg twice daily for 3 days and then 0.6 mg daily for 12 days	Standard care	Mortality, rate of intubation, rate of hospital discharge
**Mareev et al.**	2021	NRCT	Hospitalized COVID-19 adult patients	21/22	1 mg colchicine during the first 1–3 days followed by 0.5 mg/day	Placebo	Mortality, length of hospital stay.
**Brunetti et al.**	2020	Cohort	Hospitalized COVID-19 adult patients	33/33	loading dose: 1.2 mg. maintenance dose: 0.6 mg twice daily	Standard care	Mortality, hospital discharge, clinical improvement
**Scarsi et al.**	2020	Cohort	Hospitalized COVID-19 adult patients	122/140	colchicine 1 mg/day (reduced to 0.5 mg/day, if severe diarrhoea)	Standard care	survival

*RCT* Randomized controlled clinical trial, *NRCT* Nonrandomized controlled clinical trial

### Mortality

**Fig. 2:**
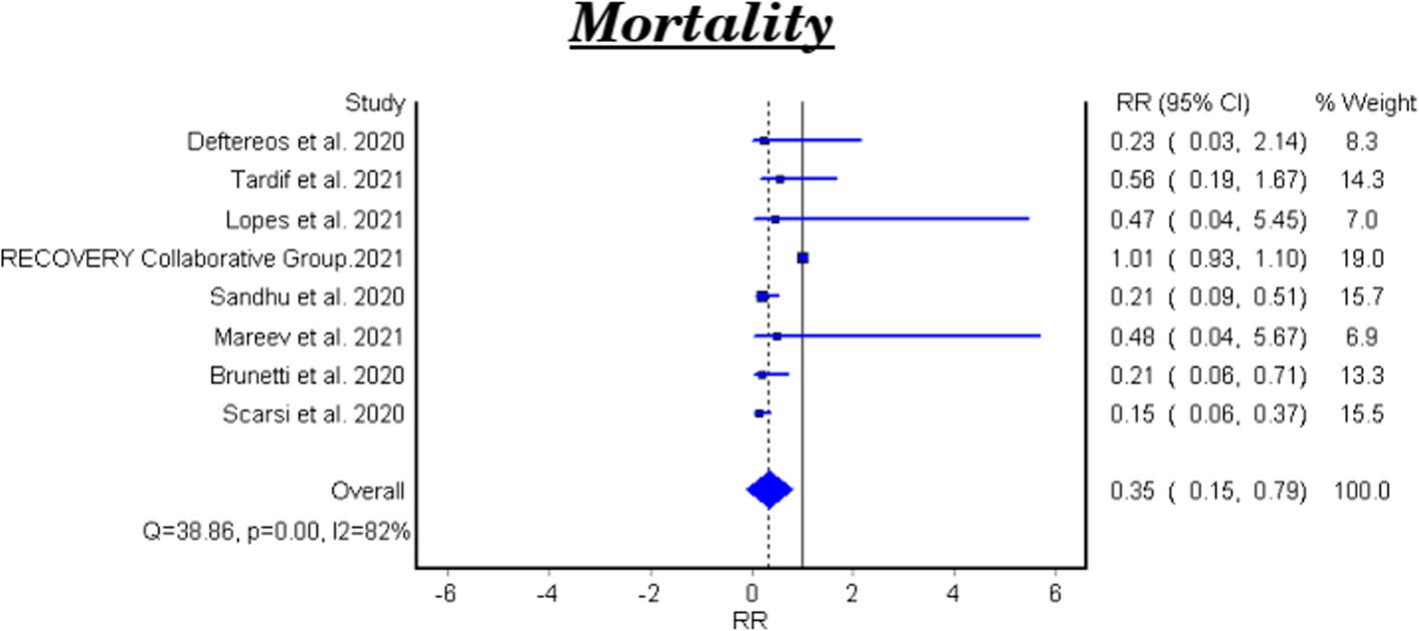
Forest plot for the efficacy of colchicine on mortality in patients with COVID-19

Table 2 and Fig. 2 showed that the meta-analysis of all included studies showed a significant difference in mortality between the treatment group with colchicine and the control group (RR 0.35, 95% CI: 0.15–0.79). There is significant heterogeneity among the studies (Homogeneity Test X2: 42.219, *P*-value < 0.000).

**Table 2:** Meta-analysis for the efficacy of colchicine on mortality in patients with COVID-19

Study	Type of study	Relative Risk	95% CI	*P*-value
Deftereos et al. 2020	RCT	0.23	0.03–2.14	0.163144
Tardif et al. 2021	RCT	0.56	0.19–1.67	0.081
Lopes et al. 2021	RCT	0.47	0.04–5.45	0.555346
RECOVERY Collaborative Group 2021	RCT	1.01	0.93–1.10	0.77
Sandhu et al. 2020	NRCT	0.21	0.09–0.51	0.00032
Mareev et al. 2021	NRCT	0.48	0.04–5.67	0.57753
Brunetti et al. 2020	Cohort	0.21	0.06–0.71	0.012
Scarsi et al. 2020	Cohort	0.151	0.062–0.368	< 0.0001
**Fixed Effect Model**		0.911	0.827–1.005	---
**Random Effect Model**		**0.35**	**0.15–0.79**	

*95% CI* 95% confidence interval, *RCT* Randomized controlled clinical trial, *NRCT* Nonrandomized controlled clinical trial.

The meta-analytical result of the six clinical trials was insignificant between the treatment and control groups (RR 0.48, 95% CI 0.22–1.07). There is significant heterogeneity among the studies (Homogeneity Test X2: 11.562, *P*-value: 0.000). The meta-analytical result of the two cohort studies was significant between the treatment and control groups (RR 0.17, 95%CI 0.08–0.35).

### Duration of COVID-19 illness till recovery

Table 3 shows the efficacy of colchicine on the duration of COVID-19 illness till recovery. Lopes et al. reported that the median duration of COVID-19 illness in the treatment group with colchicine was 7 days vs 9 days in the control group (*P*-value =0.003) [25]. While Sandhu et al., and Mareev et al., demonstrated that colchicine had no significant effect on the illness duration [26, 27]. (Table 3).

**Table 3:** Efficacy of colchicine on the duration of COVID-19 illness till recovery

Study	Type of study	Median duration of illness Colchicine vs control	*P*-value
Lopes et al. 2021	RCT	7.0 vs 9.0	0.003
Sandhu et al. 2020	NRCT	10.5 vs 11	0.947
Mareev et al. 2021	NRCT	13.0 vs 17.5	0.079

*RCT* Randomized controlled clinical trial, *NRCT* Nonrandomized controlled clinical trial

### Need for hospitalization

Tardif et al., reported that colchicine did not show a significant effect on the COVID-19 patients’ need for hospitalization RR 0.79, 95% CI 0.60–1.03, P-value =0.081) [23].

### Need for O2 therapy

Lopes et al., demonstrated that colchicine use resulted in a significant decrease in the need for O2 therapy in patients with COVID-19 (RR 0.07, 95% CI 0.02–0.27, *P* = 0.000024) [25].

### Need for ICU admission

**Fig. 3:**
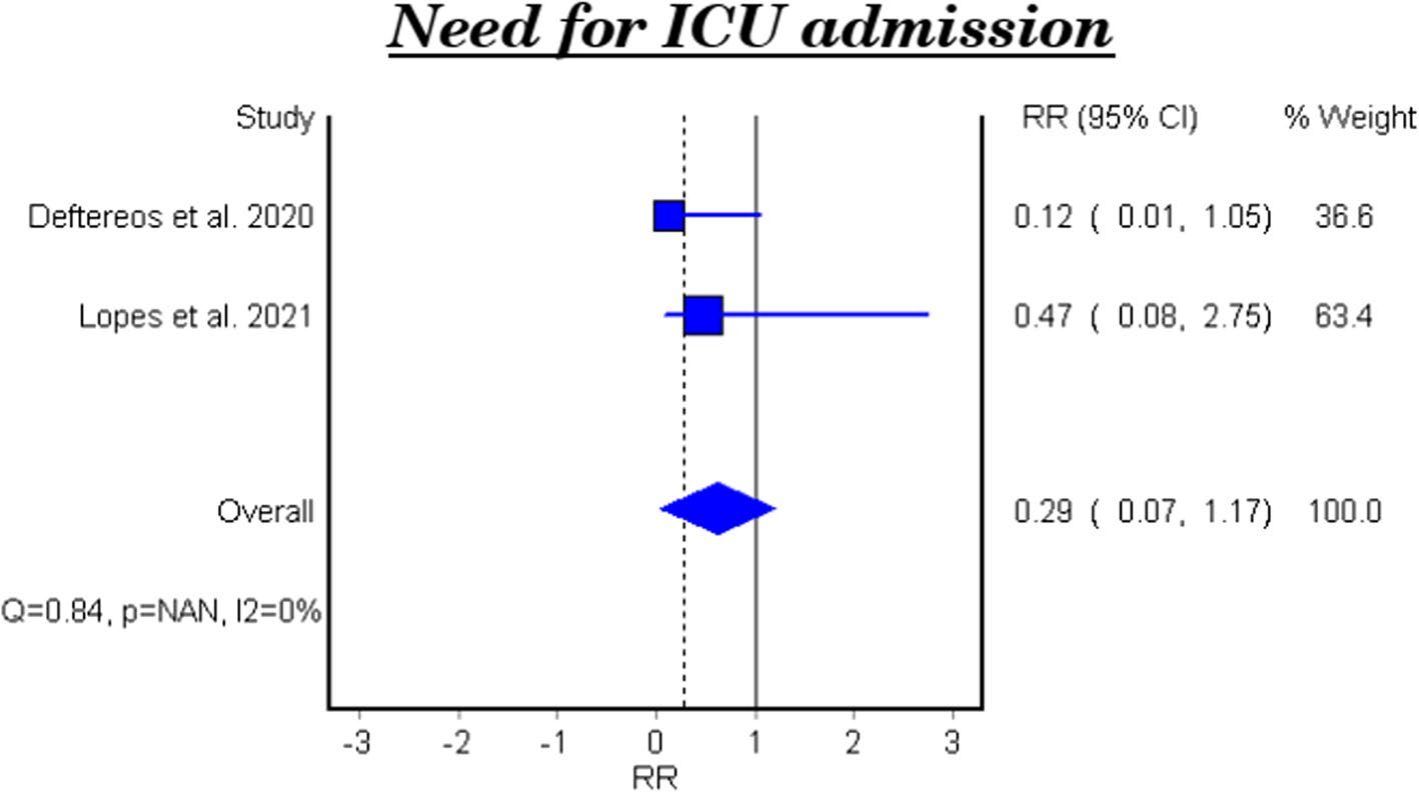
Forest plot for the efficacy of colchicine on need for ICU admission in patients with COVID-19

Table 4 and Fig. 3 show the efficacy of colchicine on need for ICU admission in patients with COVID-19. The meta-analytical result did not show a significant effect (RR 0.29, 95% CI: 0.07–1.17).

**Table 4:** Meta-analysis for the efficacy of colchicine on need for ICU admission in patients with COVID-19

Study	Type of study	Relative Risk	95% CI	*P*-value
Deftereos et al. 2020	RCT	0.12	0.01–1.05	0.025696
Lopes et al. 2021	RCT	0.47	0.08–2.75	0.393769
**Fixed Effect Model**		0.29	0.07–1.17	–

*95% CI* 95% confidence interval, *RCT* Randomized controlled clinical trial

### Need for artificial ventilation

**Fig. 4:**
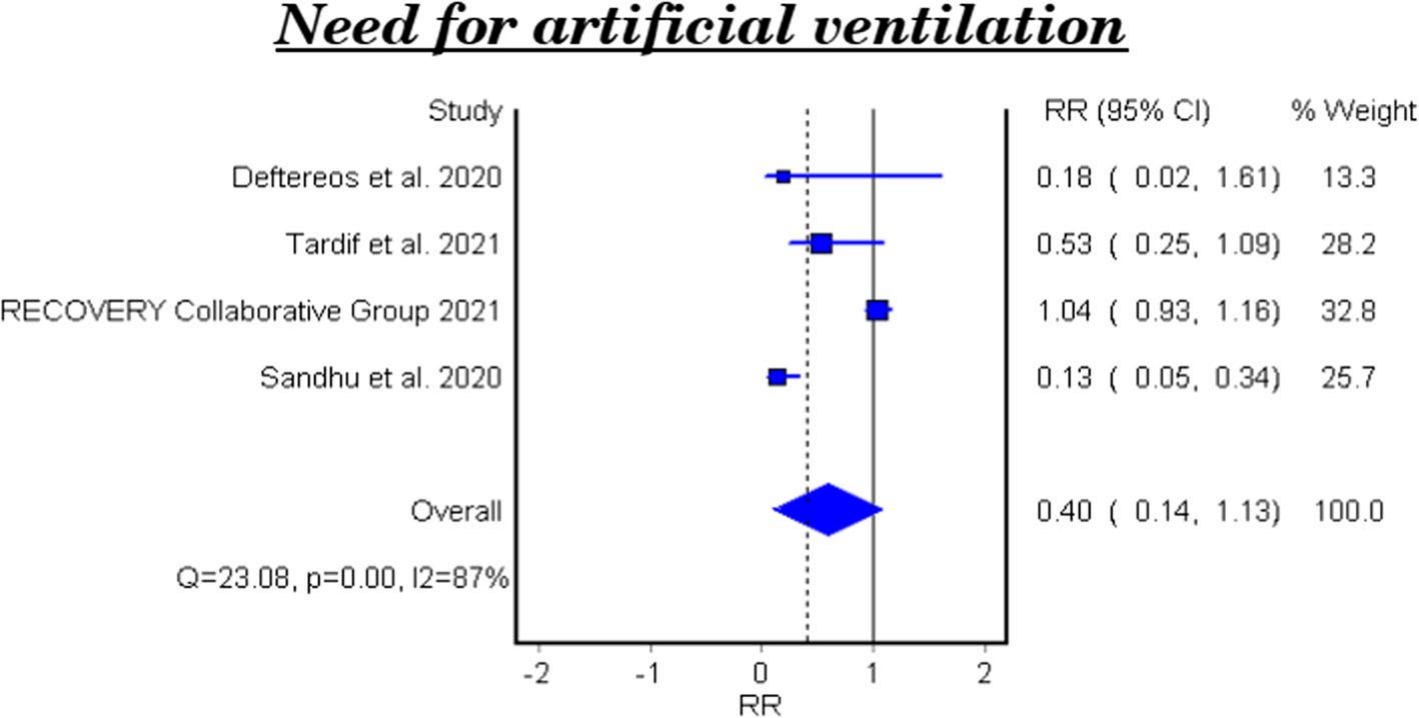
Forest plot for the efficacy of colchicine on need for artificial ventilation in patients with COVID-19

Table 5 and Fig. 4 show the efficacy of colchicine on need for artificial ventilation in patients with COVID-19. The meta-analysis of four studies demonstrated that colchicine has no significant effect on the need for artificial ventilation (RR 0.40, 95% CI 0.14–1.13). There is significant heterogeneity among the studies (Homogeneity Test X2: 18.417, *P*-value: 0.000).

**Table 5:** Meta-analysis of the efficacy of colchicine on need for artificial ventilation in patients with COVID-19

Study	Type of study	Relative Risk	95% CI	*P*-value
Deftereos et al. 2020	RCT	0.18	0.02–1.61	0.088555
Tardif et al. 2021	RCT	0.53	0·25–1.09	0·081
Recovery Collaborative Group 2021	RCT	1.04	0.93–1.16	0·48
Sandhu et al. 2020	NRCT	0.13	0.05–0.34	< 0.00001
**Fixed Effect Model**		0.992	0.874–1.126	---
**Random Effect Model**		**0.40**	**0.14–1.13**	
**Homogeneity Test** X^2^: 18.417 *P* Value: 0.000				

*95% CI* 95% confidence interval, *RCT* Randomized controlled clinical trial, *NRCT* Nonrandomized controlled clinical trial

### Hospital discharge rate

**Fig. 5:**
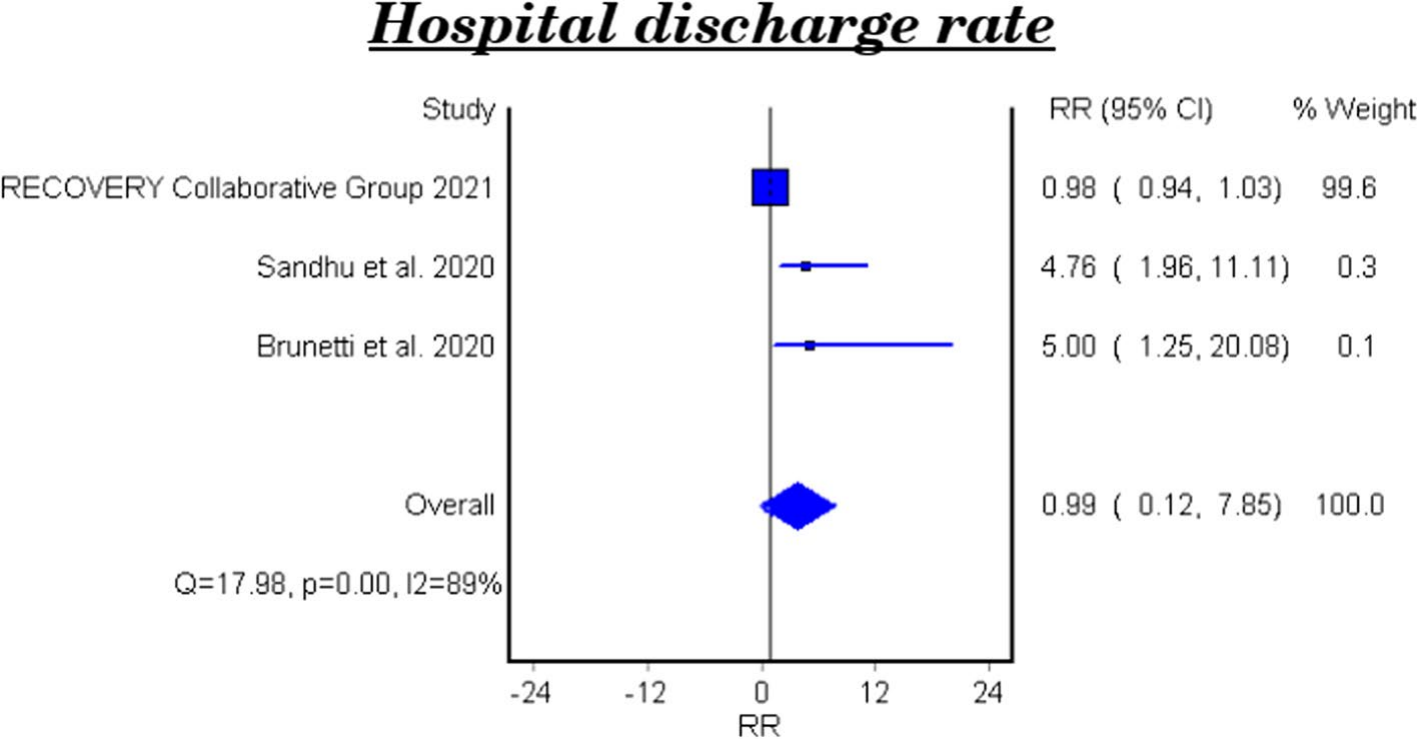
Forest plot for the efficacy of colchicine on hospital discharge rate in patients with COVID-19

Table 6 and Fig. 5 show the efficacy of colchicine on hospital discharge rate in patients with COVID-19. The meta-analytical result of the three studies demonstrated that colchicine did not show a significant effect on the hospital discharge rate (RR 0.99, 95%CI 0.12–7.85).

**Table 6:** Meta-analysis for the efficacy of colchicine on hospital discharge rate in patients with COVID-19

Study	Type of study	Relative Risk	95% CI	*P*-value
Recovery Collaborative Group 2021	RCT	0.98	0.94–1.03	0·44
Sandhu et al. 2020	NRCT	4.76	1.96–11.11	0.00032
Brunetti et al. 2020	Cohort	5.0	1.25–20.08	0.023
**Fixed Effect Model**		**0.99**	**0.12–7.85**	–

*95% CI* 95% confidence interval, *RCT* Randomized controlled trial, *NRCT* Nonrandomized controlled trial

The effect of colchicine on the hospital discharge rate in the clinical trials was not significant (RR 0.98, 95%CI 0.12–8.02), while a cohort study reported that colchicine showed a significant effect on the hospital discharge rate (RR 5.0, 95%CI 1.25–20.08, P-value 0.023) [28].

## Subgroup analysis among PCR confirmed COVID-19 patients

### Mortality among PCR confirmed COVID-19 patients

**Fig. 6:**
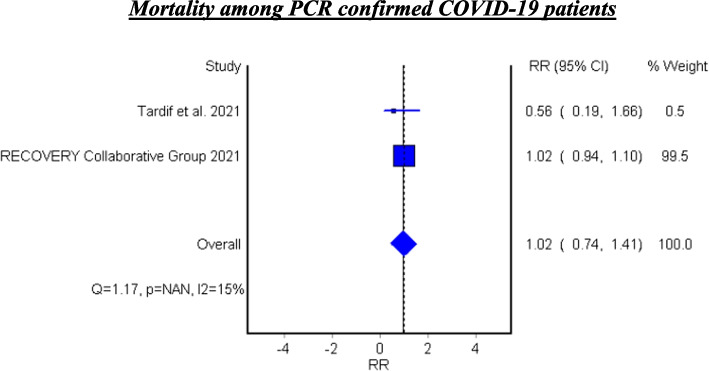
Forest plot for the efficacy of colchicine on mortality among PCR confirmed COVID-19 patients

Table 7 and Fig. 6 show the efficacy of colchicine on mortality among PCR confirmed COVID-19 Patients. Colchicine did not show a significant effect on mortality among PCR confirmed COVID-19 patients (RR 1.02, 95% CI 0.74–1.41).

**Table 7:** Meta-analysis for the efficacy of colchicine on mortality among PCR confirmed COVID-19 Patients

Study	Type of study	Relative Risk	95% CI	*P*-value
Tardif et al. 2021	RCT	0.56	0.19–1.66	0.042
Recovery Collaborative Group 2021	RCT	1.02	0.94–1.10	0.70
**Fixed Effect Model**		**1.02**	**0.74–1.41**	–

*95% CI* 95% confidence interval, *RCT* Randomized controlled trial

#### Mortality among PCR confirmed COVID-19 patients

See Fig. 6.

#### Hospitalization among PCR confirmed COVID-19 patients

Tardif et al. assessed the efficacy of colchicine on hospitalization and reported that colchicine resulted in decreased hospitalization among the PCR confirmed COVID-19 patients (RR 0.75, 95%CI 0.57–0.99, P 0.042) [23].

#### Mechanical ventilation among PCR confirmed COVID-19 patients

Tardif et al. found that colchicine has no significant effect on mechanical ventilation among PCR confirmed COVID-19 Patients (RR 0.50, 95%CI 0.23–1.07, P 0.042) [23].

## Discussion

In this meta-analysis, the studies investigated the role of colchicine in the management of COVID-19 were reviewed.

After a comprehensive search, eight studies were identified. Two of them were cohort studies (Brunetti et al., and Scarsi et al.) while the other studies were four randomized control trials (Deftereos et al., Recovery Collaborative Group, Lopes et al., and Tardif et al.) and two non-randomized trials (Mareev et al., and Sandhu et al.). The current meta-analysis involved 16,488 patients; 8146 were in the treatment group who received colchicine and 8342 were in the control group who received a placebo or standard treatment [20–27].

### The efficacy of colchicine on mortality

The eight pooled studies evaluated the efficacy of colchicine on mortality among COVID-19 patients and showed a significant reduction in the mortality rate among patients received colchicine in comparison with placebo or standard care. This result coincides with the findings of a recent systematic review that reported a significant decrease in the all-cause mortality in three observational studies [28]. In addition, a recently published meta-analysis reported that colchicine resulted in decreased mortality among COVID-19 patients. This study pooled four randomized control trials and five observational studies and involved 5522 patients only [29].

On the other hand, Mehta, et al. and Toro-Huamanchumo, et al. documented that colchicine had no effect on the mortality rate among COVID-19 patients [30, 31].

The heterogeneity test between the pooled studies showed a significant difference, which indicates interstudy variation. Pooling of these heterogeneous studies added more useful information.

According to our result, colchicine may have a beneficial effect to decrease mortality among COVID-19 patients. It was obvious that this effect occurred when colchicine was used within the early days of the disease. These findings can be explained by the anti-inflammatory role of colchicine that is mediated through the interaction between colchicine and microtubules which play an important role in cellular division, migration, and adhesion. This effect robustly influences the immune system response and reduces the inflammatory reaction. Also, colchicine decreases the release of cytokines and inflammatory mediators that stimulate the immune cells [32].

The subgroup analysis of the two cohort studies demonstrated a significant effect of colchicine on mortality among COVID-19 patients. However, the subgroup analysis for the six clinical trials showed that colchicine has no effect on mortality in the management of COVID-19. This result is consistent with the pooled analysis of a recent study where four clinical trials only were included [33]. This variation could be attributed to difference of the study design, variation in follow up duration and the colchicine regimen used in these studies.

### The efficacy of colchicine on the duration of COVID-19 illness till recovery

The efficacy of colchicine on the duration of COVID-19 illness was assessed in three clinical trials. Lopes et al. found that hospitalized COVID-19 patients who received colchicine had a shorter duration of illness till recovery in comparison with the patients who received placebo [23]. This is similar to the result reported by a recent study [34]. This finding can be related to the anti-inflammatory and immune modulatory roles of colchicine in the management of COVID-19. On the other hand, two clinical trials reported that colchicine did not affect the duration of COVID-19 illness [23, 25]. These findings agree with the results of a recently published study investigated the efficacy of colchicine on the duration of COVID-19 clinical course [31].

### The efficacy of colchicine on need for hospitalization

Tardif et al., investigated the efficacy of colchicine among non-hospitalized COVID-19 patients vs placebo. They found that colchicine did not influence the need for hospitalization among the non-hospitalized patients [21]. A recent clinical trial was conducted to assess the effect of colchicine on the prognosis of non-hospitalized COVID-19 patients and the results showed no significant effect of colchicine on hospitalization rate of the patients [35].

### The efficacy of colchicine on need for O2 therapy

Lopes et al., assessed the efficacy of colchicine on the need for O2 therapy and the results demonstrated that colchicine use resulted in a significant decrease in the need for O2 therapy in patients with COVID-19 [23]. This result can be understood based on the beneficial effect of colchicine on the inflammatory response.

### The efficacy of colchicine on need for ICU admission

The pooled results of two clinical trials showed that colchicine did not improve the need of ICU admission compared to placebo or standard care. This finding is concomitant with a recent study that included six studies only [30].

### The efficacy of colchicine on need for artificial ventilation

Four pooled studies evaluated the efficacy of colchicine on need for artificial ventilation and showed that colchicine did not decrease the need for artificial ventilation compared to placebo or standard care [20–22, 24].

The heterogeneity test between the pooled studies regarding the need for artificial ventilation showed a significant difference, which indicates interstudy variation.

This can be attributed to the variation of duration and dose of colchicine regimens in these studies, and the severity of the disease. Tardif et al., included non-hospitalized COVID-19 patients while the other three studies involved hospitalized patients.

### The efficacy of colchicine on hospital discharge rate

Three pooled studies evaluated the efficacy of colchicine on hospital discharge rate and showed that colchicine did not improve the hospital discharge rate in comparison with placebo or standard treatment [22, 24, 26].

Furthermore, the subgroup analysis of the pooled results included two clinical trials and showed that colchicine did not cause a significant improvement in the hospital discharge rate compared to placebo or standard treatment [22, 24]. On the other hand, the cohort study demonstrated a beneficial effect of colchicine on the hospital discharge rate compared to standard care [26].

The variation of the results of the three studies could be attributed to the difference of study design, number of included patients, and the treatment regimens used.

### Subgroup analysis among PCR confirmed COVID-19 patients

Two pooled studies evaluated the efficacy of colchicine among PCR confirmed COVID-19 patients and showed that colchicine did not significantly decrease mortality among PCR confirmed patients [21, 22].

In addition, Tardif et al. assessed the efficacy of colchicine on hospitalization rate among PCR confirmed COVID-19 patients and found that colchicine significantly decreased the hospitalization rate compared to placebo. Also, Tardif et al. evaluated the effectiveness of colchicine on mechanical ventilation rate among PCR confirmed COVID-19 patients and showed no beneficial effect of colchicine on mechanical ventilation in comparison with placebo [21].

## Conclusion

The study demonstrates that colchicine administration leads to a notable reduction in mortality rates and a decrease in the necessity for oxygen therapy among individuals with COVID-19. Although its impact on broader outcomes like hospitalization rates, ICU admissions, and discharge rates remains minimal, there’s a significant finding regarding its efficacy in lowering hospitalizations specifically among PCR-confirmed COVID-19 patients. This detailed understanding highlights the potential of colchicine as a therapeutic intervention for COVID-19, particularly in mitigating mortality risks and oxygen therapy requirements. These results offer valuable insights for clinicians, highlighting the need to consider colchicine as a viable treatment option for COVID-19 patients, while also emphasizing the necessity for further exploration to optimize its clinical utility.

## Data Availability

Our study is a Systematic Review/Meta-analysis. The datasets analyzed during the current study are available in the published pooled study. Also, the datasets used and analyzed during the current study available from the corresponding author on reasonable request.
